# Community perceptions of targeted anti-malarial mass drug administrations in two provinces in Vietnam: a quantitative survey

**DOI:** 10.1186/s12936-016-1662-2

**Published:** 2017-01-06

**Authors:** Thuy-Nhien Nguyen, Pham N. Huong Thu, Ngo Trong Hung, Do Hung Son, Nguyen Thanh Tien, Nguyen Van Dung, Huynh Hong Quang, Lorenz von Seidlein, Phaik Yeong Cheah, Arjen M. Dondorp, Nicholas P. J. Day, Nicholas J. White, Tran Tinh Hien

**Affiliations:** 1 Oxford University Clinical Research Unit, Wellcome Trust Major Oversea Programme, Ho Chi Minh City, Vietnam; 2 Binh Phuoc Malaria Prevent and Control Center, Binh Phuoc, Vietnam; 3 Institute of Malariology-Parasitology and Entomology (IMPE), Qui Nhon, Vietnam; 4 Mahidol Oxford Tropical Medicine Research Unit, Faculty of Tropical Medicine, Mahidol University, Bangkok, Thailand; 5 Centre for Tropical Medicine and Global Health, Nuffield Department of Medicine, Churchill Hospital, Oxford, UK

**Keywords:** Malaria, *Plasmodium falciparum*, *Plasmodium vivax*, Mass drug administration, South-East Asia, Vietnam, Knowledge, Attitude, Perceptions, Quantitative survey

## Abstract

**Background:**

As part of a targeted malaria elimination project, mass drug administrations (MDAs) were conducted in Vietnam. The impact of MDAs on malaria transmission depends largely on the efficacy of the anti-malarial drug regimen, the malaria epidemiology in the site and the population coverage. To explore why some people participate in MDAs and others do not, a quantitative survey of the villagers’ perceptions was undertaken in Vietnam.

**Methods:**

In 2013/2014 MDAs were conducted in a village in Binh Phuoc province and a village in Ninh Thuan province. Within three months of the drug administration, 59 respondents in a village in Binh Phuoc and 79 respondents in a village in Ninh Thuan were randomly selected and interviewed.

**Results:**

Comprehension of the purpose of the intervention was of paramount importance for participation in the intervention. Respondents aware that the intervention aims to protect against malaria were significantly more likely to participate than respondents who were unaware of the MDA’s purpose. Secondly, how and by whom villagers were informed was critical for participation. There was a strong association between sensitization by an informant such as a member of the local health team with participation in the intervention.

**Conclusions:**

The study suggests several approaches to increase participation in mass drug administration campaigns. Training trustworthy informants to sensitize the study population is critical to maximize village participation in this setting. To achieve high coverage the entire community must understand and agree with the intervention.

**Electronic supplementary material:**

The online version of this article (doi:10.1186/s12936-016-1662-2) contains supplementary material, which is available to authorized users.

## Background

With the growing threat of multidrug resistance in the Greater Mekong subregion, the international malaria community is recognizing the need for additional measures, including mass drug administrations (MDAs) as a component of rapid malaria elimination efforts [1]. Although their popularity has gone through cycles, MDAs have been used for malaria control and elimination for more than a century [2]. The impact of MDAs on malaria transmission is variable [3]. Effectiveness depends on the efficacy of the anti-malarial drug regimen and the proportion of the population participating [4]. The duration of the impact depends to a large part on local malaria epidemiology. While there is an extensive literature on the efficacy of anti-malarial drugs, information on how to achieve maximum coverage is limited.

A recent literature review found 28 detailed descriptions of community engagement in anti-malarial mass administrations over the last 100 years [5]. Despite the heterogeneity in populations, community engagement and study methods, the authors identified several commonalities. The top-down approach based on hierarchical structures such as government, village leaders and village elders was traditionally relied-on to mobilize the populations. The use of authority which for example could be relied on during smallpox eradication campaigns [6] has become less popular and less successful. Instead investigators depend more on a bottom-up approach based on the targeted community itself [7]. The authors of the review concluded that both approaches top-down and bottom-up are essential for success [5]. The most successful campaigns invested in a two-pronged approach by engaging the leaders of the targeted communities as well as the community members themselves. A better understanding what makes a successful campaign would be helpful for the design of future campaigns.

As part of a targeted malaria elimination (TME) project, MDAs were conducted in two villages in Vietnam. The aim of the present study was to identify factors associated with MDA participation among a random sample of community members in two villages.

## Methods

### Study site

**Fig. 1:**
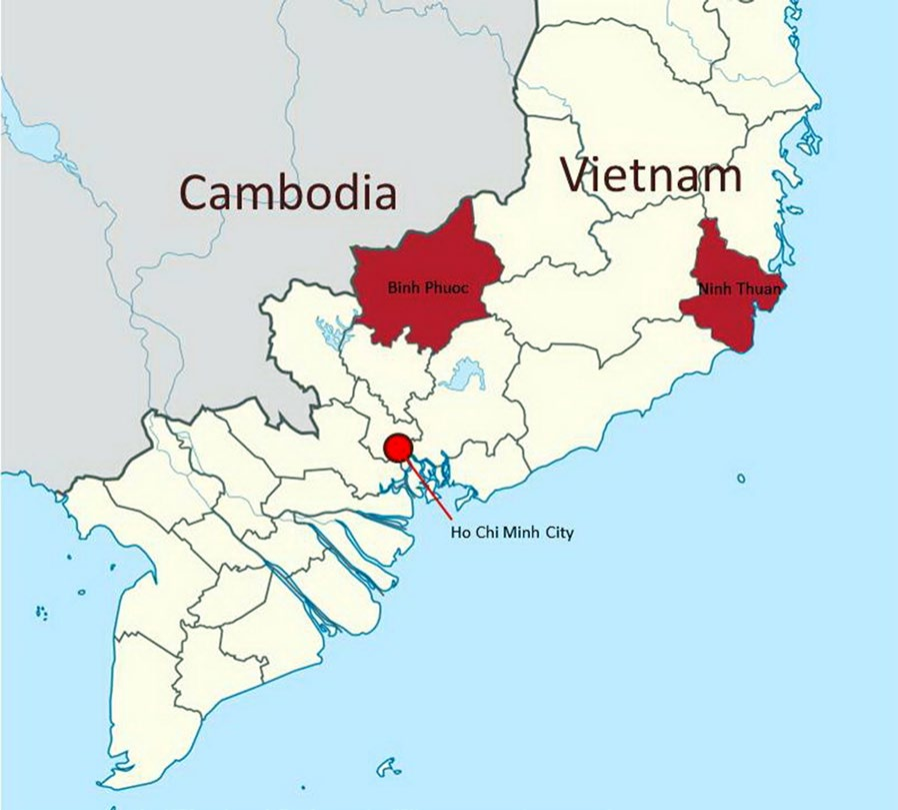
Map of Vietnam indicating the location of the two provinces (Binh Phuoc and Ninh Thuan) where the study was conducted

Despite a substantial reduction in the incidence of malaria over the last twenty years in Vietnam the disease remains a public health challenge. Since 2010, studies in Binh Phuoc province show an increased proportion of slow-clearing artemisinin-resistant infections [8], but cure rates using ACT (dihydroartemisinin–piperaquine) are still satisfactory in 2014 [9]. Two villages in Dak O commune, Binh Phuoc province and two villages in Phuoc ha Commune, Ninh Thuan province were selected based on parasite prevalence, enthusiasm of villagers to participate, and access (road conditions) (Fig. 1). Restricted randomization was used to determine which villages in each province received the intervention, MDA in the first year and which village would serve as control. The control villages also received MDA after one year of surveillance but no interviews were conducted in control villages. The names and precise co-ordinates of the study villages are withheld by the authors to protect the confidentiality of the study participants. The MDAs were carried-out in collaboration with the malaria control programme of Vietnam, the Institute of Malariology, Parasitology, and Entomology (IMPE), Ho Chi Minh City and IMPE, Qui Nhon.

### Community engagement

**Fig. 2:**
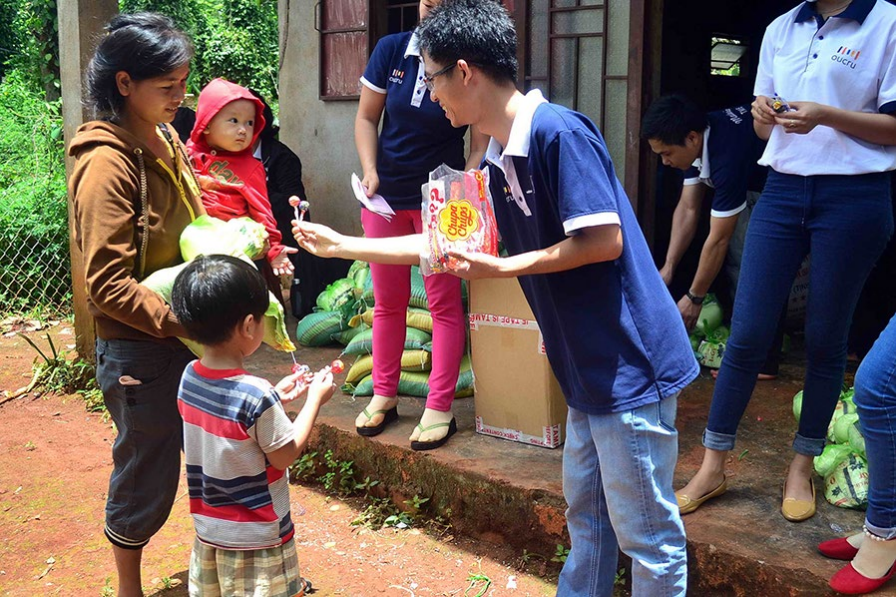
Examples of community engagement; house to house visits to inform residents about the planned campaign. ©Nguyen Thuy-Nhien

**Fig. 3:**
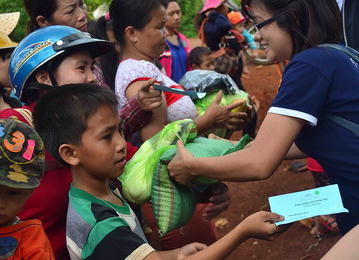
Examples of community engagement; distribution of rice to study participants. ©Nguyen Thuy-Nhien

**Fig. 4:**
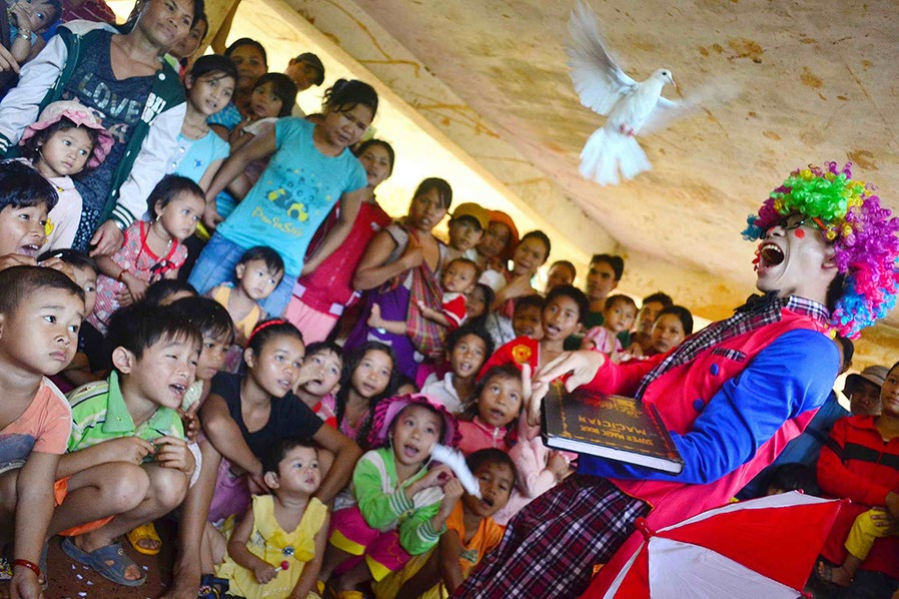
Examples of community engagement; children entertainment during a community meeting. ©Nguyen Thuy-Nhien

The project was discussed with representatives of the health authority, sequentially at the provincial, district, commune and village level. Village meetings were convened by the local study investigators, the commune health workers and the Peoples Committee staff to plan the intervention and to obtain consent from the villagers. This was followed by house-to-house visits by study investigators and the community health workers to explain the purpose of the campaigns. The commune health workers are living in villages in malaria endemic areas and are trained in malaria case detection and management. The commune health workers received additional, specific training before the study was conducted to be able to provide adequate information on malaria to the participants. Banners and posters were used to advertise the campaigns. The campaign was also announced on local radio stations. Participants received non-monetary incentives such as sweets and rice. Elements of the community engagement campaign are illustrated in three photographs (Figs. 2, 3, 4).

### The mass drug administration

**Fig. 5:**
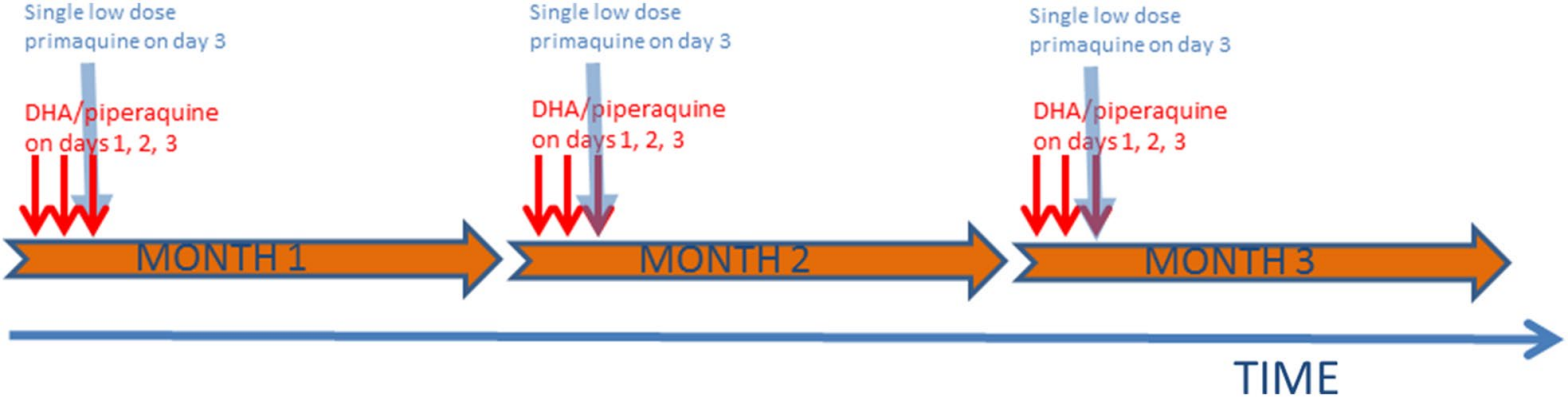
A schematic representation of treatment regimen in targeted malaria elimination (TME). Each drug administration consisted of three rounds, 1 month apart, of three doses of DHA/piperaquine. A single, low dose primaquine was administered with each round of anti-malarial drugs

The rational and the methodology for the mass drug administrations have recently been described [4, 10]. The drug regimen comprised three rounds of anti-malarial drugs one month apart (Fig. 5). Each round consisted of three daily doses of DHA/piperaquine combined with a single low dose primaquine (15 mg or 0.25 mg/kg). Each resident was encouraged to take part in three rounds, which constituted a total of nine DHA/piperaquine doses over the course of three months plus a single dose of primaquine at each round based on the following rationale. DHA remains a powerful, short-acting anti-malarial effective against most *P. falciparum* strains. Piperaquine mops up surviving parasites and prevents re-infection for the following 30 days, at which time the next round of TME is administered [11, 12]. Three doses DHA/piperaquine are needed to clear a *Plasmodium falciparum* infection. A minimum of three rounds is thought to be needed to interrupt malaria transmission, as infectious mosquitoes may survive 30 days and infect previously treated people. Furthermore, some community members may be absent during the drug administration and are thus treated during subsequent rounds [13–16]. A single low dose primaquine is sufficient to clear rapidly gametocytes, which are not susceptible to schizontocidal drugs [15]. All drugs were administered under direct observation by the study staff at a central location. The drug administrations were embedded within several malaria elimination strategies including community engagement, improved case management, and vector control strategies.

### Data collection

A questionnaire used in a survey following a MDA in West Africa was adapted to the local context and translated into Vietnamese [17]. The interview guide and questionnaire are included in the Additional file 2. The instrument included a set of questions that explored opinions and knowledge related to malaria and its control. Interviewers were trained to administer the instrument in a neutral fashion to minimise bias towards preferred responses. At both villages, the interviews were completed within three months of the completion of the MDAs. A sample of participants and non-participants were randomly selected from the database collected during the MDA. Due to an expected correlation in the answers from multiple members of a household only one person was interviewed per household. To be eligible, he or she had to be over 18 years of age, residing in the village at the time of the MDA and consent to be interviewed. A study physician who had neither participated in the engagement campaign nor the drug administration conducted the interviews.

### Data management and analysis

The questionnaires were single-entered into a database and checked for consistency. Inconsistent data were verified and corrected. The data were merged with a dataset recording the participation in the MDA. Residents who took zero MDA doses are defined for the purposes of the analysis as “non-participants”, residents who took at least one but less than nine doses are defined as “partial-participants”, and residents who took all nine doses are defined as “full-participants”. Interviewees are referred to as respondents. The administration of a single low dose primaquine with the third dose DHA/piperaquine during each round was not included in this analysis. In the initial analysis, socio-economic and demographic characteristics were explored to explain differences in degrees of participation. Comparisons of categorical data were made using Fisher’s exact or Pearson Chi squared test as appropriate. Continuous data were compared using Student’s *t* test or in the case of more than two categories, with Kruskal–Wallis equality-of-populations rank test. Considering the large number of variables and hypotheses tested only a conservative p-value <0.01 was considered significant. A logistic regression model was used to test the association between predisposing variables and the outcome (non-participant vs. participant i.e. ≥1 dose anti-malarials). Terms that appeared thematically relevant and/or were significant in the univariate analysis were explored in the model. In the final model, only variables significant below p < 0.01 were retained, namely literacy, knowledge of the causes of malaria, recall of being informed about the MDA, who explained the MDA, comprehension of the rationale and finally the purpose for the MDA. Statistical analyses were performed using Stata 14.1 (StataCorp LP, College Station, TX, USA).

## Results

The MDAs were conducted from November 2013 to January 2014 in Binh Phuoc and from January 2014 to March 2014 in Ninh Thuan. The interviews were conducted in Binh Phuoc in February and March of 2014 and in Ninh Thuan in May and June 2014. In Binh Phuoc 55 participants and 55 non-participants were invited to join the interview and in Ninh Thuan 57 participants and 39 non-participants. Of the 206, 138 agreed to be interviewed (59 in Binh Phuoc and 79 in Ninh Thuan). 69 (50%) had taken the complete drug regimen of 9 doses, 22 (16%) participated but did not take the entire course and 47 (34%) respondents did not take any dose at all. In the village in Binh Phuoc 17 of 59 respondents (29%) and in Ninh Thuan 30 of 79 respondents (38%) did not participate at all in the MDA. The majority of respondents in Ninh Tuan (48/79; 61%) had participated in all three rounds (9 doses) and 36% (21/59) in Binh Phuoc (p = 0.097). There was a statistically significant difference in the number respondents who only took a partial course 21/59 (36%) in Binh Phuoc in contrast to only 1/79 (1%) in Ninh Thuan (p < 0.0001; Table 1). The study explored the association between a range of demographic variables with participation (Table 1). There was a significant association between residency (having always lived in the study village), ethnic group (Raglai and Kinh), literacy and religion with degree of participation in the MDA. Whereas age group, marital status, occupation, and having children did not seem to influence participation.

**Table 1:** Characteristics of the 138 respondents

	Number (%) by participation				p value
	Non	Partial	Full	Total	
	0 doses	1–8 doses	9 doses		
n	47 (34%)	22 (16%)	69 (50%)	138 (100%)	
Village					<0.001
VN10 Binh Phuoc	17 (29%)	21 (36%)	21 (36%)	59 (100%)	
VN 30 Ninh Thuan	30 (38%)	1 (1%)	48 (61%)	79 (100%)	
Residency					<0.001
Always lived in study village	37 (33%)	12 (11%)	64 (57%)	113 (100%)	
Relocated into study village	9 (38%)	10 (42%)	5 (21%)	24 (100%)	
NA	1 (100%)	0	0	1 (100%)	
Median age (IQR)	32.5 (28–46)	35.5 (27.5–48.5)	39.0 (27–48)	35.0 (27–48)	0.528
Ethnicity					<0.001
Raglai	30 (39%)	1 (1%)	46 (60%)	77 (100%)	
S’Tieng	8 (22%)	11 (30%)	18 (49%)	37 (100%)	
Kinh	9 (41%)	8 (36%)	5 (23%)	22 (100%)	
Others	0 (0%)	2 (100%)	0 (0%)	2 (100%)	
Marital status					0.108
Married	36 (31%)	19 (16%)	61 (53%)	116 (100%)	
Single	7 (44%)	1 (6%)	8 (50%)	16 (100%)	
Widow/er	3 (100%)	0 (0%)	0 (0%)	3 (100%)	
NA	1(33%)	2 (67%)	0	3 (100%)	
Can read and write?					0.007
Yes	18 (24%)	16 (21%)	42 (55%)	76 (100%)	
No	27 (47%)	4 (7%)	27 (46%)	58 (100%)	
NA	2 (50%)	2 (50%)	0	4 (100%)	
Occupation					0.168
Farmer	36 (31%)	16 (14%)	63 (55%)	115 (100%)	
Labourer	5 (46%)	4 (36%)	2 (18%)	16 (100%)	
Manufacturing	1 (50%)	1 (50%)	0 (0%)	2 (100%)	
Professional	2 (50%)	0 (0%)	2 (50%)	4 (100%)	
Retired	1 (100%)	0 (0%)	0 (0%)	1 (100%)	
Trader	0 (0%)	0 (0%)	2 (100%)	2 (100%)	
NA	2 (67%)	1 (33%)	0	3 (100%)	
Religion					0.004
None	35 (39%)	5 (6%)	50 (56%)	90 (100%)	
Christian	4 (18%)	7 (32%)	11 (50%)	22 (100%)	
Buddhist	5 (36%)	4 (29%)	5 (36%)	14 (100%)	
Ancestor worship	2 (29%)	3 (43%)	2 (29%)	7 (100%)	
No response	0 (0%)	3 (75%)	1 (25%)	4 (100%)	
Other	1 (0%)	0 (0%)	0 (0%)	1 (100%)	
Do you have children?					0.762
Yes	37 (33%)	18 (16%)	59 (52%)	114 (100%)	
No	9 (39%)	4 (17%)	10 (44%)	23 (100%)	
NA	1 (100%)	0	0	1 (100%)	

*NA* no answer/not available, *IQR* inter quartile range

**Fig. 6:**
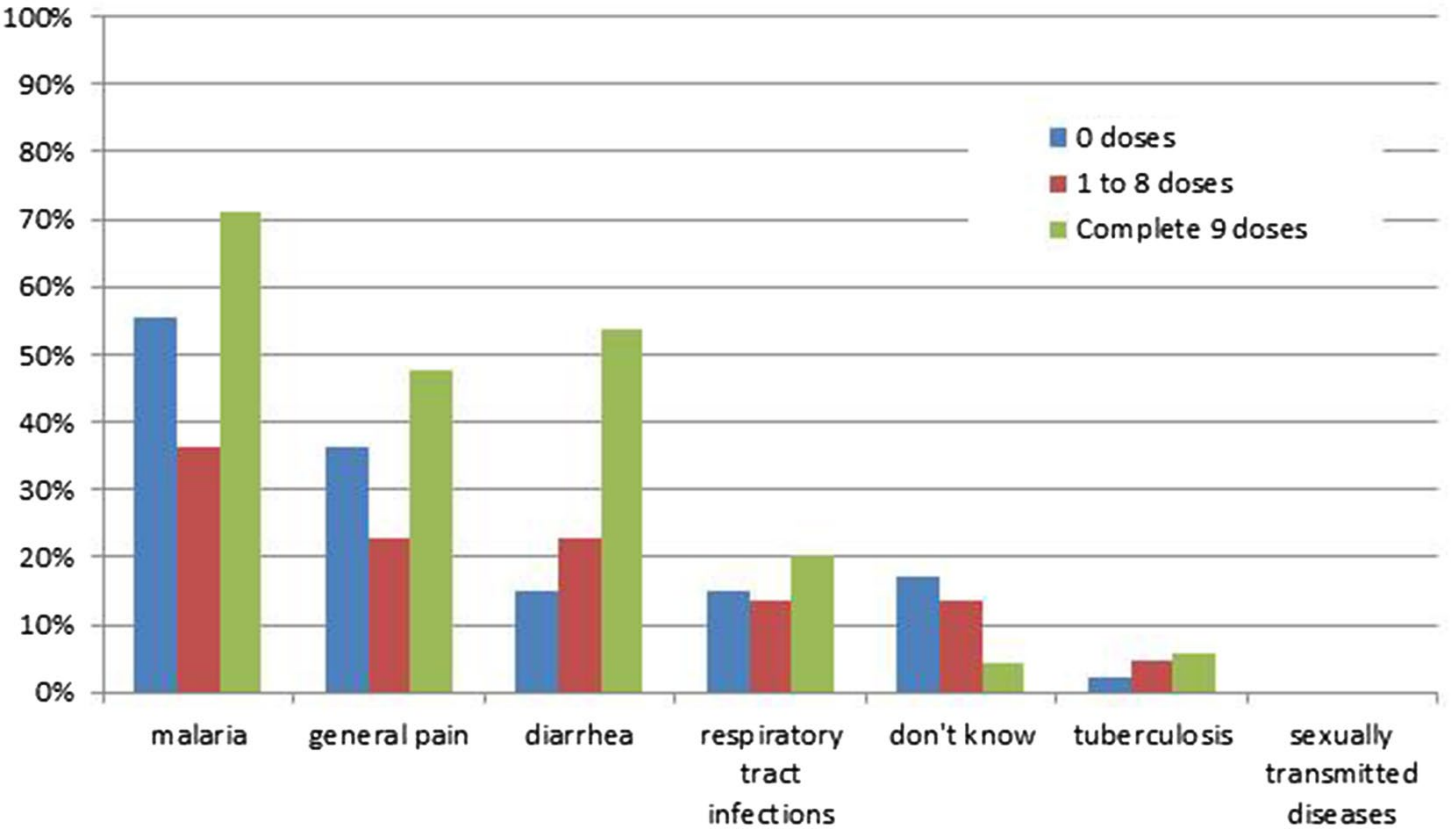
Perception of which diseases cause the most health problems in the village, by the number of doses ingested (more than one response was permitted)

Respondents were asked which diseases cause the most health problems in their village. The relationship between the perception which diseases cause the most health problems in the village and participation is illustrated in Fig. 6. 49/69 (71%) of the respondents who took the full course of 9 doses stated that malaria causes most problems in the village followed by diarrhoea, general pain and respiratory tract infections. There was general agreement that relative to the other diseases, malaria caused the most health problems (p = 0.449) but the percentage who thought that malaria was the biggest problem was lower among the partial- 8/22 (36%) and non-participants 26/47 (55%) compared with the full participants (p = 0.491).

**Fig. 7:**
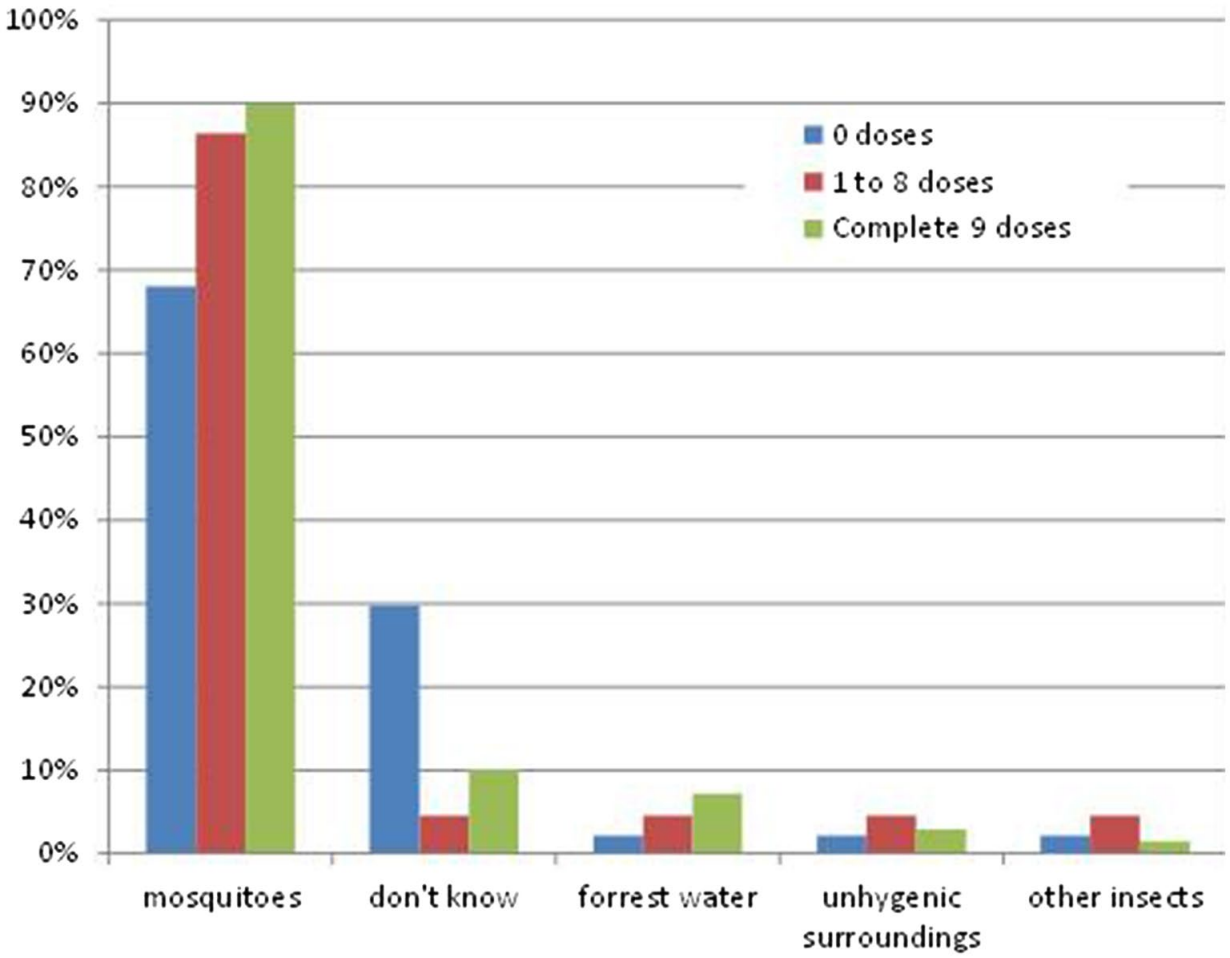
Perceptions of what causes malaria, by the number of doses ingested (more than one response was permitted)

Independent of participation status, over 90% of the respondents stated that they used bed nets to prevent malaria. Keeping the household clean, insecticides and mosquito coils were less frequently mentioned. Partial-participants were more likely to mention cleanliness or insecticides compared to non- and full-participants (Additional file 1: Figure S1). The most frequently mentioned symptoms associated with malaria were in decreasing order headache, shivering and fever (Additional file 1: Figure S2). 6/47 (13%) non-participants mentioned fever as a symptom of malaria in contrast to 20/69 (29%) full-participants (p = 0.119). When asked about the causes of malaria the non-participants were less likely to respond that mosquitoes transmit malaria (p = 0.010) and more likely to respond “don’t know” (p = 0.005) compared to partial- and full participants (Fig. 7).

**Fig. 8:**
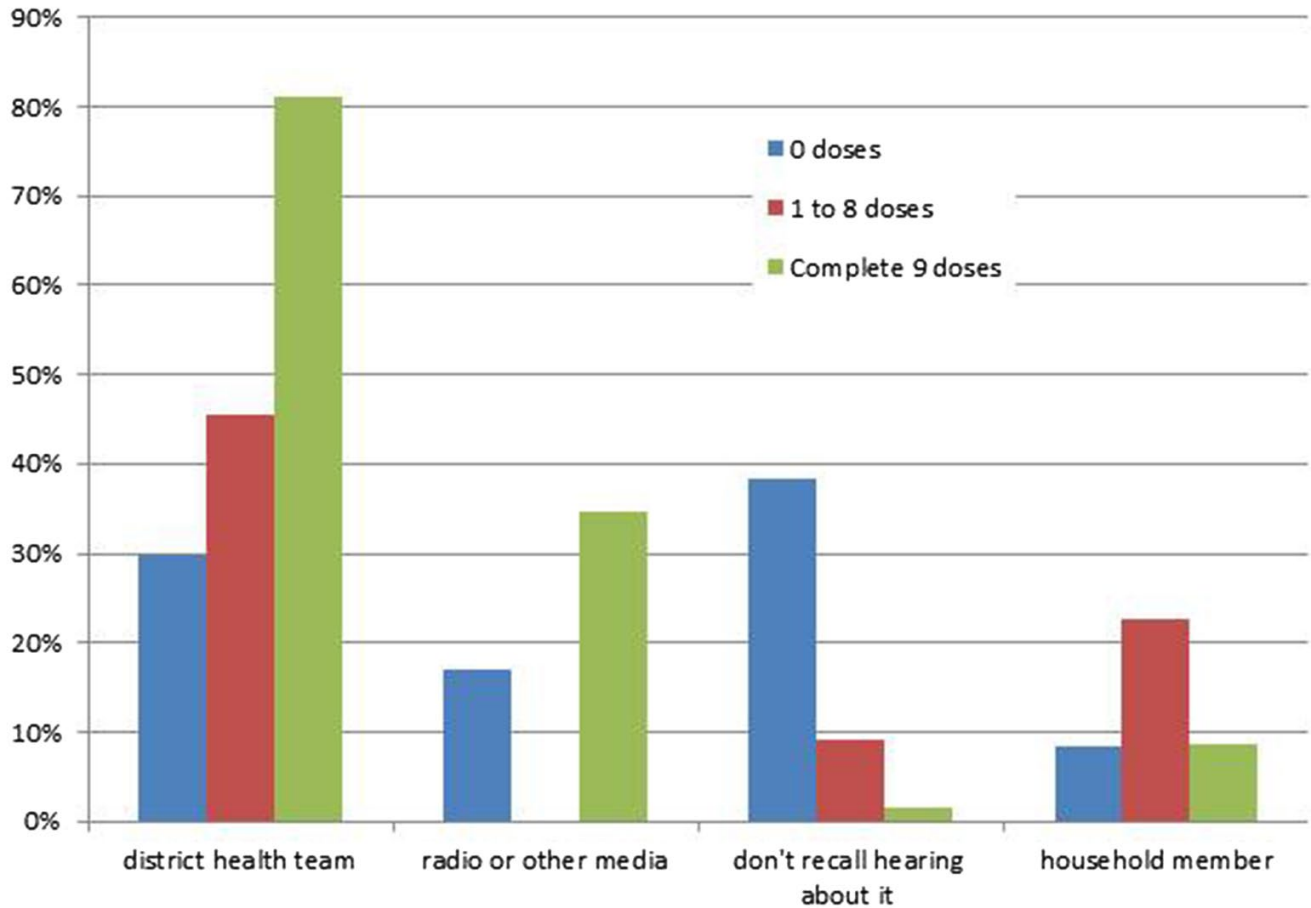
Response to how he/she heard about the MDA, by the number of doses ingested (more than one response was permitted)

Non-participants were significantly less likely to respond that they heard about the MDA from the district health team (p < 0.001) and more likely to respond that they could not recall if they were informed (p = 0.001) than partial- and full participants (Fig. 8). None of the respondents reported to have received information about the MDAs by the banners, flyers or through other villagers.

**Fig. 9:**
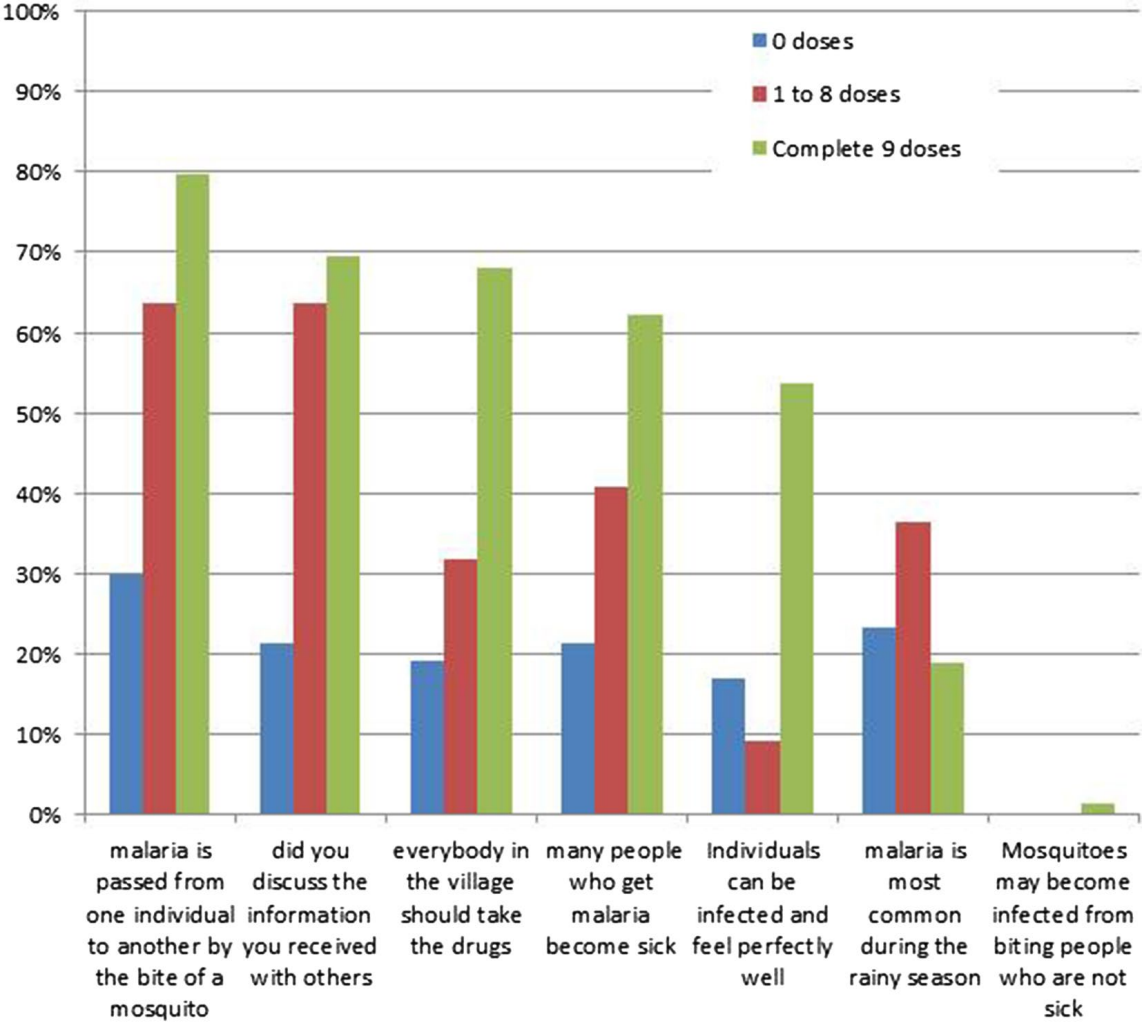
Response to what he/she understood about the MDA, by the number of doses ingested (more than one response was permitted)

Important concepts regarding the transmission of malaria were understood by a much larger percentage of full-participants compared to non-participants. For example, 55/69 (80%) respondents who took the complete course agreed that malaria is transmitted by mosquitoes from one individual to another in contrast to only 14/47 (30%) non-participants (p < 0.001). The critical concept of subclinical parasite infections was understood by only about half of the full participants 37/69 (54%) and even fewer (8/47 or 17%) of the non-participants (p = 0.009; Fig. 9). The large majority of respondents did not believe that mosquitoes become infected from biting infected people who were asymptomatic.

**Fig. 10:**
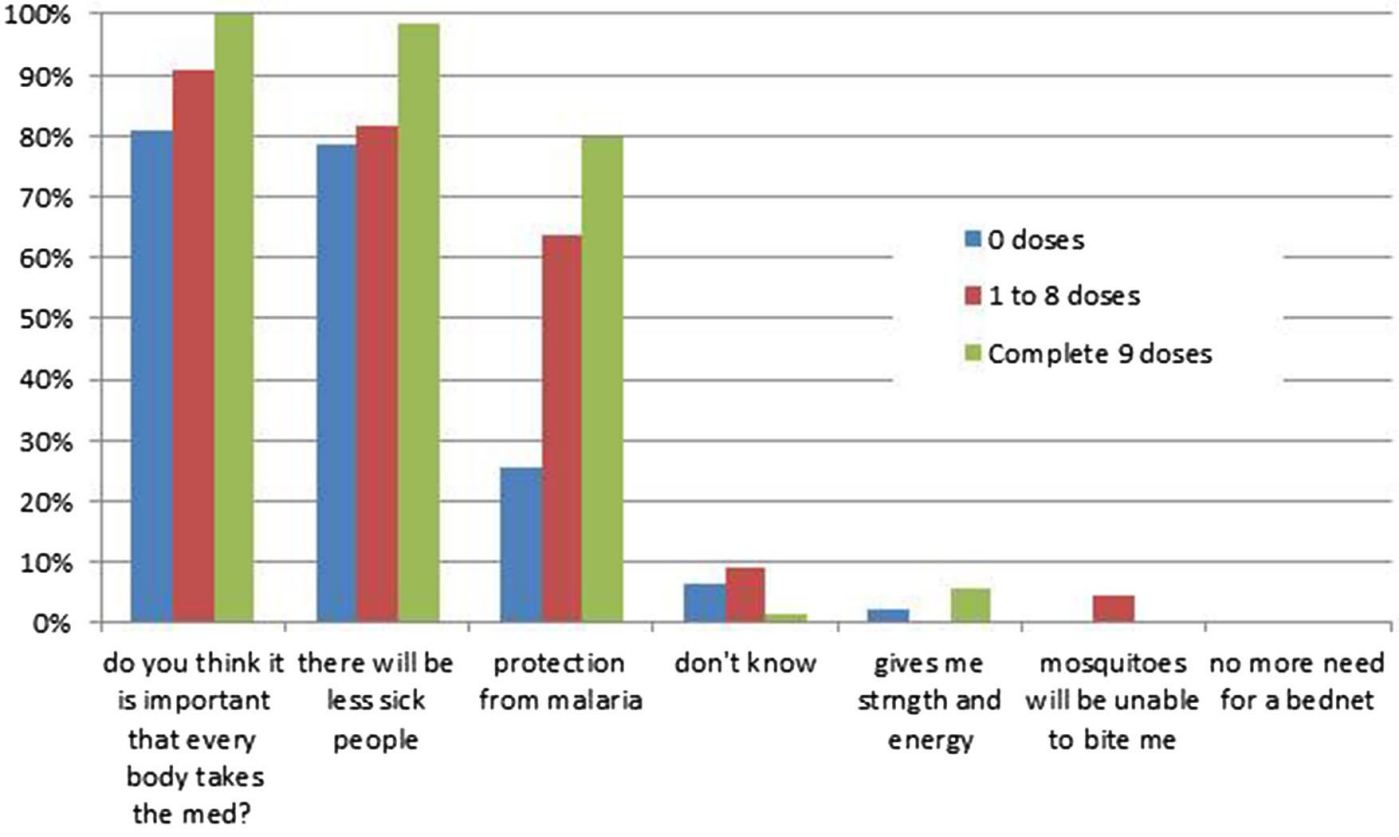
Response to what he/she thinks the medicine given during the MDA is for by the number of doses ingested (more than one response was permitted)

The message that it is important that everyone in the village takes the anti-malarial drugs had been understood by over 80% of the respondents irrespective of the number of doses taken. A similar percentage thought there would be less malaria following the campaign. The concept that the campaign could protect against malaria was accepted by 80% (55/69) of the respondents who took the complete course but only 12/46 (26%) of the non-participants (p < 0.0001). Importantly, none of the respondents felt that the anti-malarial drugs should replace the need for a bed net (Fig. 10).

**Fig. 11:**
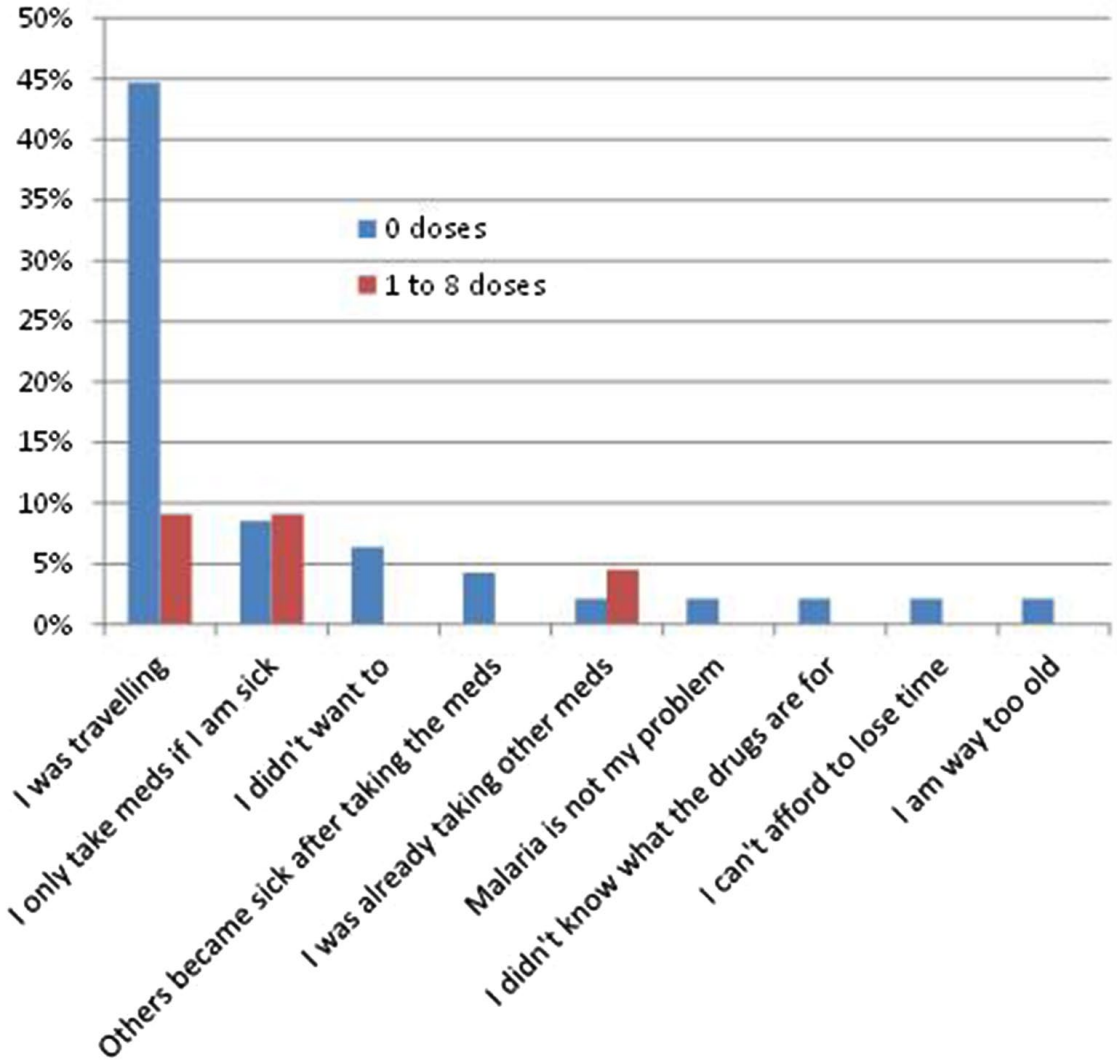
Response of non-participants to why he/she did not take the medicine, by the number of doses ingested (more than one response was permitted)

Non- and partial-participants were asked why they didn’t participate. As shown in Fig. 11 by far the most frequent reported reason for non-participation was travel.

A univariate analysis of 15 variables found that 6 were highly significantly associated with participation in the MDA (Table 2). Only one variable was found to be independently and significantly associated with participation. Whether participants could recall being informed about the MDA remained highly significant after adjusting for other variables. This remained the single independently significant variable in an alternative model when comparing full-participants with partial- and non-participants.

**Table 2:** Adjusted odds ratios of 138 respondents for variables associated with participation in the MDA

	Non-participants	Partial and full participants	OR crude	p value	OR adj*	p value
n	47 (34%)	91 (66%)				
Sex (male)	34 (37%)	58 (63%)	1.488	0.311	2.580	0.082
Ethnicity (S’Tieng)	8 (22%)	29 (78%)	2.280	0.066	4.594	0.016
Village (VN30)	17 (29%)	42 (72%)	1.513	0.262	2.808	0.098
Median age in years (IQR)	34 (28–46)	37 (27–48)	1.007	0.632	1.022	0.252
Occupation (Farmer)	36 (31%)	79 (69%)	1.795	0.235	1.635	0.428
Literacy	18 (24%)	58 (76%)	2.807	0.006	1.400	0.506
Religion (Christian)	42 (34%)	82 (66%)	1.085	0.890	1.497	0.616
Recent immigration	34 (32%)	72 (68%)	1.449	0.372	1.560	0.469
Believes that malaria is the village’s main health problem	26 (31%)	57 (69%)	1.354	0.406	0.739	0.567
Believes fever is a symptom of malaria	6 (21%)	23 (79%)	2.311	0.093	1.691	0.394
Doesn’t know causes of malaria	33 (29%)	83 (72%)	4.402	0.002	1.593	0.481
Doesn’t recall being told about the MDA	22 (21%)	84 (79%)	13.636	<0.001	7.083	0.001
Who explained MDA (DHT)	11 (15%)	63 (85%)	7.364	<0.001	1.401	0.648
Believes everybody should participate in the MDA	8 (18%)	37 (82%)	3.340	0.006	0.965	0.955
Believes the medicine protects against malaria	12 (15%)	69 (85%)	8.886	<0.001	2.364	0.194

Non-participants are defined as not having taking any doses of the antimalarial drug (DHA/piperaquine). Participants have taken at least one or more doses

adj* adjusted for the statistically significant terms in the crude odds ratio [Literacy, Doesn’t know causes of malaria, Doesn’t recall being told about the MDA, Who explained MDA (DHT), Believes everybody should participate in the MDA, Believes the medicine protects against malaria]

*OR* odds ration, *DHT* district health team

## Discussion

The study found that the pivotal difference between participation and non-participation in the MDA was the recollection of being adequately informed about the campaign. Villagers who recalled being informed about the campaign were much more likely to participate than those who did not. Specifically, residents who had been explained about the MDA by the local health team were significantly more likely to complete the entire course of the drug administration. More qualitative interviews would be needed to explore whether trust or multiple factors beyond trust influenced these decisions. A detailed and locally-appropriate explanation of anti-malarial drug administration campaigns is needed for an understanding of the complex concepts of malaria transmission, the role of subclinical infections in malaria transmission and ultimately the acceptance of interventions to interrupt transmission. Demographics also played a role in participation; village residency, older age, ethnicity, religion and literacy were associated with participation. In contrast, the occupation of the respondents (most of whom were farmers) and whether they had children did not make a significant difference in participation.

This study relied on recollection and opinions which may be biased and inaccurate. This is illustrated by the stated reason for non-participation. Nearly 45% of the non-participants said that they were travelling at the time of the MDA. When untrue, this response may have allowed the respondent and the interviewers to “save face”; to spare the interviewer, as well as the respondent, the embarrassment of stating the real reason for non-participation. Although none of the respondents suggested absence of trust in the researchers or a dislike for the drugs as reasons for non-participation, this does not necessarily exclude such perceptions. To get a more detailed understanding of the true reasons for incomplete or non-participation including deeper motivations, fears and apprehension, in-depth interviews and focus group discussions will be needed [18, 19]. A second limitation of this study is that the interviews were conducted only in two villages after MDA and with a limited number of respondents. A larger number of respondents would potentially increase the generalizability of the findings. Nevertheless, the study had sufficient statistical power to detect differences between full, partial, and non-participation.

The findings underscore the importance of community mobilization prior to drug administration campaigns and could inform how campaigns can be implemented in an effective way to maximize participation. The study provides evidence about the importance not only of what information is disseminated but where the information comes from. Messages, which made an impact, came from a trusted familiar source of heath information. It may be necessary to invest time and money to establish such core information providers to sensitize the entire community appropriately long before an anti-malarial drug administration campaign is undertaken. Research is under way to better understand which means of communication to explain the underlying concepts and purpose of MDA. It is also important to identify demographic strata that are less likely to participate and special efforts should be undertaken to engage this subgroup of the community. In the study villages, it would have been worthwhile to specifically visit and engage members of two ethnic groups, Raglai and Kinh. The demographic data also suggest that it may be worthwhile to take extra efforts to include younger, less educated and more recently arrived members of the community to treat all members of the community.

The findings from this study are consistent with recently published findings from a quantitative study following four mass administrations of anti-malarial drugs along Thai-Myanmar border areas [20]. While the findings in two of the four villages were comparable to the findings reported here the other two villages had issues which resulted in fragmented communities suggesting that a cohesive community is a helpful if not essential predisposition for successful mass drug administrations. Several qualitative and quantitative studies following anti-malarial mass administrations were conducted in The Gambia, West Africa. There the researchers found travel, perceived adverse drug reactions and rumours, inconveniences related to the logistics of MDA (e.g. waiting times) and the perceived lack of information about MDA were critical reasons for non-participation [17, 21, 22]. While the research into factors related to the participation in mass administrations of anti-malarial drugs is somewhat limited there is a broad experience how to engage communities in other biomedical interventions including interventions against the transmission of HIV, tuberculosis, and vector-borne disease [23]. This body of work has resulted in a framework for community engagement in global health research which has applicability for MDAs. Lavery and co-workers suggest twelve points to consider for effective community engagement. The provision of information and the building of trust feature prominently in the framework suggesting some universal principals which have to be respected for successful community engagement [24].

## Conclusions

The elimination of malaria poses large challenges as all community members, not only high risk groups have to participate in interventions. The findings from this study suggest several approaches to maximize participation in mass drug administration campaigns and thereby contribute to a broader understanding what makes community engagement successful. The concepts underlying anti-malarial mass administration are complex and need time to be explained especially if the target population has only a primary education or less. In the absence of a detailed understanding of the rationale the residents in the target villages must be able to trust the people providing information about the campaign. Training and investing into the establishment of a trustworthy team to sensitize the study population may be critical to maximize village participation in this setting. To achieve high coverage the purpose of the intervention must be understood by the entire community.

## Additional files

**Additional file 1: Figure S1.** Comparison of what respondents do to prevent malaria with number of doses ingested. **Figure S2.** What kind of complaints do people with malaria have?

**Additional file 2.** Questionnaire.
