# Supplementary material for: Community perceptions of targeted anti-malarial mass drug administrations in two provinces in Vietnam: a quantitative survey
Source: Malar J. 2017 Jan 6;16:17. doi: 10.1186/s12936-016-1662-2 (PMC5216593; doi:10.1186/s12936-016-1662-2)

Supplementary materials

Figure S1: Comparison of what respondents do to prevent malaria with number of doses ingested

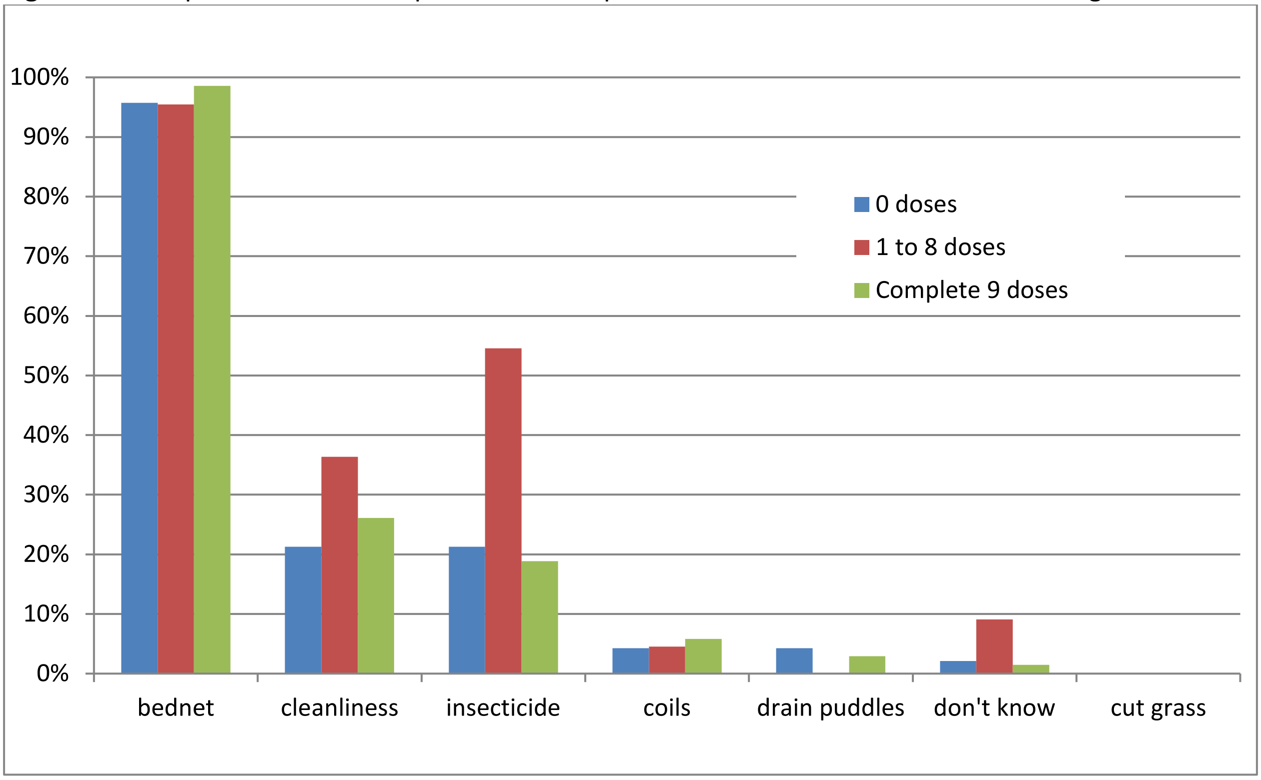

Figure S2: What kind of complaints do people with malaria have?

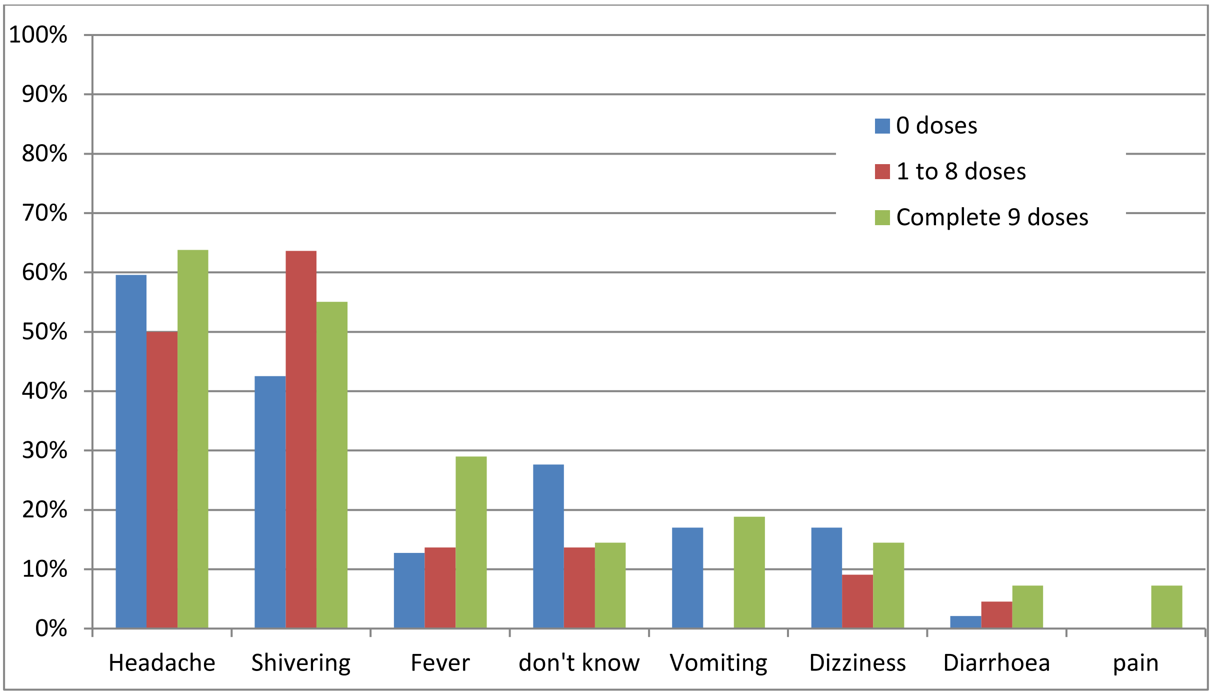

Supplement: Supplementary file 1 — Additional file 1: Figure S1. Comparison of what respondents do to prevent malaria with number of doses ingested. Figure S2. What kind of complaints do people with malaria have? [file 12936_2016_1662_MOESM1_ESM.pdf]
