# Supplementary material for: Community perceptions of targeted anti-malarial mass drug administrations in two provinces in Vietnam: a quantitative survey
Source: Malar J. 2017 Jan 6;16:17. doi: 10.1186/s12936-016-1662-2 (PMC5216593; doi:10.1186/s12936-016-1662-2)
Supplement: Supplementary file 2 — Additional file 2. Questionnaire. [file 12936_2016_1662_MOESM2_ESM.pdf]

# Survey on Community Perceptions of Targeted Malaria Elimination

**Content**
- **Interview guide**
- **Questionnaire**

**Interview Guide for survey on community perceptions**

**Whom to interview?**

The aim of the survey is to find out why some people participate in the campaign and others don't. Are there factors we could modify to allow all people to participate? Children are unable to provide helpful answers, instead the parent or an adult guardian of the child is interviewed. There could be considerable similarities of answers coming from the same household – therefore we recommend interviewers to only interview one respondent per household. The investigators locate respondents with the help of the data manager who can produce a list of all participants and non-participants. If there is a limited number of non-participants (<50) in a village it is reasonable to target all of them. If there are more than 50 non-participants people for interviews are selected using random methods.

**How to interview?**

One of the most important concerns in conducting this survey is that the interviewers must have been trained very well as it requires several crucial skills:

i. Translating technical or medical terms into local languages that are as close to the original as possible.
ii. Creating informal and comfortable atmosphere while conducting the survey

Observing reactions or interpreting non-verbal expressions of the interviewees and make "appropriate" judgement on how to move on. We should make the interviewees feel comfortable from this point on:

i. By explaining why, we think their opinions and observations on a topic are important
ii. By explaining why and how they were chosen and why it is important to have their cooperation to maintain the representativeness
iii. If the informants say they don't know enough => assure them their participation is crucial and that we are truly interested in what they have to say => tell them there is no right or wrong answer
iv. By telling everyone we interview that we are trying to learn from them.

We should be careful if the interviewees give no response because it could be a sign of their discomfort, disinterest, or some negative feedbacks. If the interviewees have negative feelings towards the questionnaire and we still keep urging them to answer, this will make the interviewees frustrated and they may not want to participate in our programme again. However, no response could also be useful information as well if we interpret it along with the interviewees' facial expressions or body language. We might know to some extent what they think or how they feel with the issues being interviewed.

If staff who informed the villagers about MDA will be the ones who will conduct this interview it could be that, consciously or not, the interviewers try to influence the response. This could lead to the kind of answers that will not reflect the interviewees' understanding. Hence, one common understanding that should be made clear at the beginning is that this questionnaire was mainly aimed to know the interviewees' existing perceptions and practices as accurate as possible, not to assess the staff's performance.

The question "Where is your place of origin?" is very important. But the way we interview this question here is also very similar to the authority's interrogation. In order to avoid this uncomfortable atmosphere, we might ask briefly first whether the interviewees are original residents or not. If not, we might ask them to *tell their stories* how come and why they moved here. Then the interviewers can fill the information given in this questionnaire form.

In some places, many people still lack basic medical knowledge and do not have an exact term to refer to malaria (some people still refer to malaria as a kind of fever). Thus, the interviewers need to make sure that they and the interviewees are referring to malaria.

<table>
<tr><td>Date of interview</td><td>|_|_|/|_|_|/|_|_|_|_|</td><td></td><td>Individual ID</td><td>|_|_|_|_|_|_|_|</td><td></td></tr>
<tr><td>Initials of Interviewer</td><td>|_|_|</td><td></td><td>Location ID</td><td>|_|_|_|_|</td><td></td></tr>
<tr><td>Researcher Present</td><td>1 Yes</td><td>2 No</td><td>Name</td><td>|_|_|_|_|_|_|_|_|<br>|_|_|_|_|_|_|_|_|</td><td></td></tr>
<tr><td>Language of Interview</td><td>1<br>3</td><td>2<br>4 English</td><td>Sex<br>Date of Birth</td><td>M Male<br>|_|_|/|_|_|/|_|_|_|_|</td><td>F Female</td></tr>
<tr><td>Number of Interview Attempts</td><td>|_|</td><td></td><td>Ethnic Group</td><td>A<br>C</td><td>B<br>O Other</td></tr>
</table>

# Survey on Community Perceptions of Targeted Malaria Elimination

## Introduction

I am doing health research in this village. I am trying to learn about the knowledge, attitudes and practices of villagers in relation to malaria and the mass drug administration of antimalarials. This will help to improve the malaria control programme in the future.

## A. Background Information / Demographics

<table>
<tr><td>A1.</td><td>Marital status (CIRCLE ONE RESPONSE)<br>1 Single<br>2 Married<br>3 Widower / Widow<br>4 Divorced or separated<br>9 No response</td></tr>
<tr><td>A2.</td><td>Can you read and write? (CIRCLE ONE RESPONSE)<br>1 Yes<br>2 No<br>9 No response</td></tr>
<tr><td>A3.</td><td>What is your occupation? (CIRCLE ALL RESPONSES)</td></tr>
<tr><td></td><td>1 Student<br>2 Farmer / Rice field<br>3 Herdsmen<br>4 Trader<br>5 Tailor<br>6 Manufacturer / Builder<br>8 Driver<br>9 Retired/ Too old to work<br>10 Professional / Civil servant<br>11 None / Unemployed<br>12 Labour (daily/seasonal/long-term).<br>9 No response<br>7 Other. A3.Specify ______________________________________________</td></tr>
<tr><td>A4.</td><td>What is your religion?</td></tr>
</table>

| | |
|---|---|
| | 1 Buddhist<br>2 Christian<br>3 Ancestor worship/traditional believes<br>4 None / Atheist<br>9 No response<br>5 Other. A5.Specify ________________________ |
| A5. | Where is your place of origin? |
| | 1 Have you been born and lived here<br>A5.1.1 If yes have you always lived in your current home? ____________<br>A5.1.2. If you have moved house why? ____________<br>2 Have you relocated<br>A5.2.1. If yes how many years ago:____________<br>A5.2.2. From where:____________<br>A5.2.3. Why: ____________<br>9 No response<br>3 Other. A3.Specify ________________________ |
| A6. | Do you have children? |
| | 1 Yes<br>2 No<br>9 No response<br>3 Other. A3.Specify ________________________ |
| | |

## B. Malaria Questionnaire (Knowledge & Practices)

| | | | | |
|---|---|---|---|---|
| B1. | What sickness causes most health problems in your village? (CIRCLE ALL RESPONSES) | | | |
| B1.1. | Diarrhoea | 1 Unprompted | 2 Prompted Yes | 3 Prompted No / DK |
| B1.2. | General body pain / Joint pain | 1 Unprompted | 2 Prompted Yes | 3 Prompted No / DK |
| B1.3. | Malaria | 1 Unprompted | 2 Prompted Yes | 3 Prompted No / DK |
| B1.4. | Respiratory tract infections (coughing etc.) | 1 Unprompted | 2 Prompted Yes | 3 Prompted No / DK |
| B1.5. | Sexually Transmitted Diseases and AIDS | 1 Unprompted | 2 Prompted Yes | 3 Prompted No / DK |
| B1.6. | Tuberculosis | 1 Unprompted | 2 Prompted Yes | 3 Prompted No / DK |
| B1.8. | Don't know | 1 Unprompted | | 3 Prompted No / DK |
| B1.9. | No response | 1 Unprompted | | 3 Prompted No / DK |
| B1.7. | Other. B1. Specify ____________________________________________ | | | |
| B2. | What do you do to prevent malaria? (CIRCLE ALL RESPONSES) | | | |
| B2.1. | Burn coils | 1 Unprompted | 2 Prompted Yes | 3 Prompted No / DK |
| B2.2. | Cut down the grass | 1 Unprompted | 2 Prompted Yes | 3 Prompted No / DK |
| B2.3. | Drain the puddles | 1 Unprompted | 2 Prompted Yes | 3 Prompted No / DK |
| B2.4. | Keep household dark | 1 Unprompted | 2 Prompted Yes | 3 Prompted No / DK |
| B2.5. | Keep household / compound / surroundings clean | 1 Unprompted | 2 Prompted Yes | 3 Prompted No / DK |
| B2.6. | Spray household with insecticide | 1 Unprompted | 2 Prompted Yes | 3 Prompted No / DK |
| B2.10. | Use bednet | 1 Unprompted | 2 Prompted Yes | 3 Prompted No / DK |
| B2.11. | Use mosquito coils | 1 Unprompted | 2 Prompted Yes | 3 Prompted No / DK |
| B2.12. | Nothing | 1 Unprompted | | 3 Prompted No / DK |
| B2.8. | Don’t know | 1 Unprompted | | 3 Prompted No / DK |
| B2.7. | Other. B3. Specify ____________________________________________ | | | |
| B3. | What kind of complaints do people with malaria have? (CIRCLE ALL RESPONSES) | | | |
| B3.1. | Diarrhoea | 1 Unprompted | 2 Prompted Yes | 3 Prompted No / DK |
| B3.2. | Dizziness | 1 Unprompted | 2 Prompted Yes | 3 Prompted No / DK |
| B3.3. | Fever | 1 Unprompted | 2 Prompted Yes | 3 Prompted No / DK |
| B3.4. | General body pain | 1 Unprompted | 2 Prompted Yes | 3 Prompted No / DK |
| B3.5. | Headache | 1 Unprompted | 2 Prompted Yes | 3 Prompted No / DK |
| B3.6. | Shivering | 1 Unprompted | 2 Prompted Yes | 3 Prompted No / DK |
| B3.10. | Vomiting | 1 Unprompted | 2 Prompted Yes | 3 Prompted No / DK |
| B3.8. | Don’t know | 1 Unprompted | | 3 Prompted No / DK |
| B3.9. | No response | 1 Unprompted | | 3 Prompted No / DK |
| B3.7. | Other. B4. Specify ____________________________________________ | | | |
| B4. | What causes malaria? (CIRCLE ALL RESPONSES)<br>1 Allah / God<br>2 Drinking too much fresh cows’ milk in the rainy season<br>3 Eating mangoes<br>4 Eating too much …… in the rainy season<br>5 Mosquitoes<br>6 Other insects<br>10 Rains<br>11 Spirits<br>12 Unhygienic surroundings<br>8 Don’t know<br>9 No response<br>7 Other. B2. Specify ____________________________________________ | | | |

## Mass Drug Administration Questionnaire

Earlier this year there was a mass drug administration (MDA) programme* in this village.
* in some places referred to “taking drugs” (to kill malaria) and “studying blood.”
It is critical to make sure that the respondent understands that we are referring to the recent MDA campaign.

| | |
|---|---|
| C1. | Did you hear about this MDA? (CIRCLE ONE RESPONSE)<br>1 Yes<br>2 No. C1. Why? ________________________________ ⇒ **Go to question C1**<br>8 Don't know<br>9 No response |
| C2. | How did you hear about the MDA? (CIRCLE ALL RESPONSES)<br>1 Sensitisation by District Health Team / Village Health Workers / Study Staff<br>2 Radio or other media<br>3 Flyers / banners/ posters<br>4 Household member / s<br>5 Neighbour<br>6 Another villager<br>8 Don’t know<br>9 No response<br>7 Other. C2. Specify ________________________________ |
| C3. | Who explained the MDA to you? (CIRCLE ONE RESPONSE)<br>1 District Health Team / Village Health Workers / Study Staff<br>2 Radio or other media<br>3 Village head / mayor<br>4 Household member / s<br>5 Neighbour / s<br>6 Another villager<br>10 No one ⇒ Go to question C8<br>8 Don't know<br>9 No response<br>7 Other. C5. Specify ________________________________ |

| | | | |
|---|---|---|---|
| C4. | What did you understand from the MDA information explained to you? (CIRCLE **ALL** RESPONSES) | | |
| C4.1. | Many people who get malaria become sick | 1 Prompted Yes | 3 Prompted No / DK |
| C4.2. | Malaria is passed from one individual to another by the bite of a mosquito | 1 Prompted Yes | 3 Prompted No / DK |
| C4.3. | Malaria is more common in the rainy season | 1 Prompted Yes | 3 Prompted No / DK |
| C4.4. | Individuals can have malaria infections and feel perfectly well | 1 Prompted Yes | 3 Prompted No / DK |
| C4.5. | Mosquitoes may become infected from biting individuals who do not get sick | 1 Prompted Yes | 3 Prompted No / DK |
| C4.4. | It is difficult to tell which individuals are carrying malaria without getting sick | 1 Prompted Yes | 3 Prompted No / DK |
| C4.10. | Everybody in the village should take the drugs | 1 Prompted Yes | 3 Prompted No / DK |
| C4.11. | Nothing | | 3 Prompted No / DK |
| C4.8. | Don’t know | | 3 Prompted No / DK |
| C4.9. | No response | | 3 Prompted No / DK |
| C4.7 | Other. C4.7. Specify ________________________________ | | |
| C5. | Did you discuss the information given to you about the MDA with any other person? (CIRCLE **ONE** RESPONSE) | | |

| | |
|---|---|
| | 1 Yes. C7. With whom? ________________________________________________<br>2 No<br>8 Don't know<br>9 No response |
| C6. | What do you think the medicine is for? (CIRCLE **ALL** RESPONSES)<br>1 Protection from malaria<br>2 Mosquitoes will not be able to bite me<br>3 After taking the medicine I will not need to sleep under my bednet<br>4 Gives me strength / energy<br>8 Don't know<br>9 No response<br>7 Other. C8. Specify _________________________________________________ |

| | | | | |
|---|---|---|---|---|
| C7. | For how long do you expect to be protected from malaria after taking the medicine? (CIRCLE **ONE** RESPONSE)<br>1 Days<br>2 Weeks<br>3 Months<br>4 Years<br>5 Only during the rainy season<br>6 not sure<br>7 Don't believe that the medicine can help protect<br>8 Don't know<br>9 No response | | | |
| C8. | Do you think the number of people who fall sick with malaria will change this year? (CIRCLE **ONE** RESPONSE)<br>1 There will be less malaria attacks<br>2 There will be more malaria attacks<br>3 Stay the same<br>8 Don't know<br>9 No response | | | |

| | | | | |
|---|---|---|---|---|
| C12. | Do you think it is important for everybody in the village to take the medicine? (CIRCLE **ONE** RESPONSE)<br>1 Yes. C12.1. Why? ____<br>2 No. C12.2. Why? ____<br>3 Conditional. C12.3. Only if ____<br>8 Don't know<br>9 No response | | | |
| C13. | How long did you have to wait to receive the medicine? (CIRCLE **ONE** RESPONSE)<br>1 acceptable<br>2 quite long<br>3 too long<br>8 Don't know<br>9 No response | | | |
| C14. | Did you take the medicine the first time you were offered it? (CIRCLE **ONE** RESPONSE)<br>1 Yes ⇒ Go to question C20<br>2 No. C18. Why? ____<br>8 Don't know<br>9 No response | | | |
| C15. | Why did you not take the medicine in the MDA? (CIRCLE **ALL** RESPONSES) | | | |
| C15.1. | I did not want to take the medicine | 1 Unprompted | 2 Prompted Yes | 3 Prompted No / DK |
| C15.2. | I was travelling / I was not in the village | 1 Unprompted | 2 Prompted Yes | 3 Prompted No / DK |
| C15.3. | I distrust the institution conducting the study / I am not happy with the institution / We are being used by the institution | 1 Unprompted | 2 Prompted Yes | 3 Prompted No / DK |
| C15.4. | I never take any medicines | 1 Unprompted | 2 Prompted Yes | 3 Prompted No / DK |
| C15.5. | I only take medicines when I am ill | 1 Unprompted | 2 Prompted Yes | 3 Prompted No / DK |
| C15.6. | Malaria is not a problem for me | 1 Unprompted | 2 Prompted Yes | 3 Prompted No / DK |
| C15.10. | I did not know what the medicine was for | 1 Unprompted | 2 Prompted Yes | 3 Prompted No / DK |
| C15.11. | Other people became sick after taking the medicine | 1 Unprompted | 2 Prompted Yes | 3 Prompted No / DK |

| | | | | |
|---|---|---|---|---|
| C15.12. | I was told not to take the medicine ⇒ Answer C15.12.1. and C15.12.2. | 1 Unprompted | 2 Prompted Yes | 3 Prompted No / DK |
| C15.13. | I can't afford to lose time / I was working | 1 Unprompted | 2 Prompted Yes | 3 Prompted No / DK |
| C15.14. | I only take traditional medicine | 1 Unprompted | 2 Prompted Yes | 3 Prompted No / DK |
| C15.15. | I am very old | 1 Unprompted | 2 Prompted Yes | 3 Prompted No / DK |
| C15.16. | I was taking another medicine at the same time | 1 Unprompted | 2 Prompted Yes | 3 Prompted No / DK |
| C15.17. | I was pregnant | 1 Unprompted | 2 Prompted Yes | 3 Prompted No / DK |
| C15. 18 | The medicine tastes disgusting | 1 Unprompted | 2 Prompted Yes | 3 Prompted No / DK |
| C15.8. | Don't know | 8 Don't know | | 3 Prompted No / DK |
| C15.9. | No response | 9 No response | | 3 Prompted No / DK |
| C15.7. | Other C15. Specify ______ | | | |

C15.12.1 Who told you not to take the medicine?*

______

* We should be very careful when asking this question as it could be seen as threatening. We have to describe our good will clearly.

C15.12.2 Why did they tell you not to take the medicine?

______

C16. If next year the drugs were given again, would you take the medicine? (CIRCLE **ONE** RESPONSE)

1 Yes. C23.1. Why? ______
2 No. C23.2. Why? ______
3 Conditional. C23.3. Only if ______
8 Don't know
9 No response

C17. In relation to how the MDA programme was carried out

C17.1. Do you think you received enough information about the MDA?

1 Yes ⇒ Go to question C35.2
2 No. C35.1. Why did you not receive enough information? ______
8 Don't know
9 No response

C17.2. Do you think the time, days, and season of administration were appropriate?

1 Yes
2 No. C17.3. Why? ______
3 I did not take the medicine
8 Don't know
9 No response

C18. Do you think the MDA programme is important?

1 Yes. C18.1. Why? ______
2 No. C18.2. Why? ______
3 Maybe. C18.3. If ______
8 Don't know
9 No response

C19. Would you recommend the MDA programme to someone else?

| | |
|---|---|
| | 1 Yes. C19.1. Why? ______________________<br>2 No. C19.2. Why? ______________________<br>3 Conditional. C19.3. Yes if ______________________<br>8 Don't know<br>9 No response |
| C20. | How do you think that the village can help in the MDA programme?<br>______________________<br>______________________<br>______________________ |

## Final Comments

______________________

______________________

______________________

## Thanks!

Thank you for answering the questions. Good-bye and have a nice day.
